# Treatment of glaucoma by prostaglandin agonists and beta‐blockers in combination directly reduces pro‐fibrotic gene expression in trabecular meshwork

**DOI:** 10.1111/jcmm.15172

**Published:** 2020-04-08

**Authors:** Sushma Tejwani, Praveen Machiraju, Archana Padmanabhan Nair, Anuprita Ghosh, Raunak Kumar Das, Arkasubhra Ghosh, Swaminathan Sethu

**Affiliations:** ^1^ Department of Glaucoma and Cataract services Narayana Nethralaya Bangalore India; ^2^ School of Bio Sciences and Technology Vellore Institute of Technology Vellore India; ^3^ GROW Research Laboratory Narayana Nethralaya Foundation Bangalore India; ^4^ Manipal Academy of Higher Education Manipal India; ^5^ Centre for Biomaterials, Cellular and Molecular Theranostics Vellore Institute of Technology Vellore India; ^6^ Singapore Eye Research Institute Singapore City Singapore

**Keywords:** beta‐blockers, fibrosis, IOP‐lowering medications, prostaglandins, trabecular meshwork

## Abstract

Prostaglandin analogues (PG), beta‐blockers (BB) or their combination (PG+BB) are used primarily to reduce the intraocular pressure (IOP) pathologically associated with glaucoma. Since, fibrosis of the trabecular meshwork (TM) is a major aetiological factor in glaucoma, we studied the effect of these drugs on fibrosis‐associated gene expression in TM of primary glaucoma patients. In the present study, TM and iris of primary open‐angle (n = 32) and angle‐closure (n = 37) glaucoma patients were obtained surgically during trabeculectomy and categorized based on the type of IOP‐lowering medications use as PG, BB or PG+BB. mRNA expression of pro‐fibrotic and anti‐fibrotic genes was quantified using qPCR in these tissues. The gene expression levels of pro‐fibrotic genes were significantly lower in PG+BB as compared to other groups. These observations and underlying signalling validated in vitro in human TM cells also showed reduced fibrotic gene and protein expression levels following PG+BB treatment. In conclusion, it is observed that PG+BB combination rather than their lone use renders a reduced fibrotic status in TM. This further suggests that IOP‐lowering medications, in combination, would also modulate fibrosis‐associated molecular changes in the TM, which may be beneficial for maintaining aqueous out‐flow mechanisms over the clinical treatment duration.

## 1 INTRODUCTION

Glaucoma is a disorder that eventually leads to optic neuropathy resulting in a compromise of visual field perception in a patient. It is the second leading cause of blindness worldwide and if left untreated can cause an irreversible loss of vision.^1^ Primary glaucoma is of two types—primary open‐angle glaucoma (POAG) and primary angle‐closure glaucoma (PACG) based on the morphology of irido‐corneal angle. In both the situations, there is an increase in the intraocular pressure (IOP) and eventually there is damage to the optic nerve, and loss of visual field. Trabecular meshwork (TM) contributes to 90% of the aqueous humour out‐flow via the conventional drainage pathway^2^ and hence is important for maintaining the IOP. The increase in resistance to conventional out‐flow due to changes in TM structure, that is pore closure resulting from aberrant extracellular matrix (ECM) deposition, plays a major role in glaucoma.^3^, ^4^, ^5^ The TM is composed of collagens, laminins, fibronectin, proteoglycans and matricellular proteins.^2^ Physiological TM ECM remodelling is orchestrated by a balanced regulation between pro‐fibrotic and anti‐fibrotic factors such as TGFβ, CTGF, fibronectin, collagens, hevin and decorin.^6^ Pro‐fibrotic events contribute to pore closure in TM,^7^, ^8^ and TGFβ has been shown to be key inducer of pore closure proteins in humans, animal models and in vitro studies.^9^, ^10^

The current management of glaucoma focuses on controlling increase in IOP by the use IOP‐lowering medications such as prostaglandin analogues (PG), beta‐blockers (BB), carbonic anhydrase inhibitors (CAI) and alpha agonists (AA). These medications function through decreasing production of aqueous humour and/or increasing out‐flow by alternative pathways.^11^, ^12^ Prostaglandin analogues and beta‐blockers reduce IOP by increasing uveo‐scleral out‐flow and by reducing the production of aqueous humour, respectively. Prostaglandin analogues and beta‐blockers are the most potent drugs in terms of IOP‐lowering capabilities and tolerability,^13^, ^14^, ^15^ hence, are the most commonly used drugs in glaucoma patients. It has been also shown that PG when used in combination with BB led to better outcomes due to additional reduction in IOP.^16^ Sustaining a functional trabecular meshwork (TM) during the management of glaucoma could improve prognosis. Hence, it would be relevant to choose IOP‐lowering medication that, in addition to reducing IOP, can also reduce fibrotic changes in the TM. Currently, there is no evidence regarding the effect of IOP‐lowering medications directly on the fibrotic status of TM in human patients. Exogenous PGs have been reported to have a role in initiating as well as modulating fibrosis in different organs such as lung, renal and cardiac tissues,^17^, ^18^, ^19^ but their exact role on human TM fibrosis remains unknown. Similarly, reports on the effect of BB on ECM remodelling in TM are rather sparse.^20^, ^21^ However, BB has been reported to have anti‐inflammatory effects and favourable ECM remodelling with reduced fibrosis in vascular tissues.^22^, ^23^ Considering that both PG and BB exert differential outcomes in different systems in terms of ECM regulation and remodelling,^24^, ^25^, ^26^, ^27^, ^28^ it is imperative to study the effect of primary IOP‐lowering medications used in glaucoma on the TM. The current study investigated the effect of prostaglandin analogues (PG), beta‐blockers (BB) or their combination (PG+BB) on the expression of fibrosis‐associated factors (TGFβ1, transforming growth factor beta 1; TGFβ2, transforming growth factor beta 2; TGFβR2, transforming growth factor beta receptor 2; CTGF, connective tissue growth factor; FN, fibronectin; LOXL2, lysyl oxidase–like 2; WNT3A, wingless‐type family member 3A; decorin, dermatan sulphate proteoglycan II—DSPG2 and hevin, secreted protein acidic and rich in cysteine‐like 1—SPARCL1) in TM of primary glaucoma patients and validated the observations in vitro. Our findings suggest the beneficial effects of combination of prostaglandin analogue and beta‐blockers on TM.

## 2 METHODS

The cross‐sectional study was approved by the institutional ethics committee. The study was conducted as per the guidelines stated by Indian Council of Medical Research and the Declaration of Helsinki. Patient information and samples were obtained following informed written consent.

### 2.1 Study population

Patients with a diagnosis of POAG or PACG that underwent trabeculectomy in a tertiary eye care centre were included in the study. All these patients underwent a complete clinical evaluation, that is visual acuity, refraction, Goldmann applanation tonometry to determine IOP, gonioscopy to determine whether angles are open or closed, optic nerve head evaluation, perimetry with Humphrey Field Analyser (HFA) and/or imaging of the optic nerve head with optical coherence tomography (OCT) to establish the glaucomatous damage. These patients were diagnosed as POAG when the IOP was high with open angles on gonioscopy and glaucomatous changes on clinical optic nerve evaluation. The changes in the nerve were substantiated with corresponding perimetric changes and/or RNFL thinning on OCT. Primary angle‐closure glaucoma (PACG) diagnosis was considered for patients that had narrow angles with signs of occlusion such as patchy pigmentation of trabecular meshwork, pigments on anterior lenticular surface, sphincter changes or synechial angle closure. These angle changes along with optic nerve damage substantiated by corresponding changes in perimetry and/or OCT were considered as PACG. All PACG patients underwent laser peripheral iridotomy followed by the medical treatment. These glaucoma patients whether POAG or PACG were subjected to glaucoma filtration surgery, that is trabeculectomy, when (a) the IOP could not be controlled with medical treatment, (b) intolerant to medical treatment or (c) the cataract surgery was indicated and the patient was already on more than one IOP‐lowering medications for that eye.

#### 2.1.1 Inclusion criteria

Patients with POAG or PACG undergoing trabeculectomy with or without cataract surgery. Further, the patients that were on IOP‐lowering medications that includes PG and/or BB for more than a week.

#### 2.1.2 Exclusion criteria

(a) Patients with secondary glaucoma or type of glaucoma other than POAG and PACG, (b) TM tissue with poor RNA yield, (c) patients with age less than 18 years and more than 85 years, (d) patients with serology positive for HIV, HBS and HCV, (e) patients who were operated without any IOP‐lowering medications or its use for less than a week and (f) patients using IOP‐lowering medications other than PG or BB, for example alpha agonist, carbonic anhydrase inhibitors or cholinergic agonists only.

#### 2.1.3 Sample collection and storage

Trabecular meshwork was obtained while performing trabecular meshwork block excision, and iris was collected while performing iridectomy during trabeculectomy procedure. The tissue samples were collected in sterile micro‐centrifuge tubes containing Ringer Lactate solution and stored in a bio‐repository at −80°C until further use.

#### 2.1.4 Classification of groups

The samples were divided into subgroups based on the types of primary glaucoma or types of IOP‐lowering medications. Based on primary glaucoma, the samples were divided into those from POAG (n = 32) or PACG (n = 37) patients. Based on the type of IOP‐lowering medications, the samples were divided into those on either prostaglandin analogues (PG, n = 14), beta‐blockers (BB, n = 28) or a combination of prostaglandin analogues and beta‐blockers (PG+BB, n = 27). Prostaglandin analogues used by the study cohort include bimatoprost, travoprost and latanoprost. All these three PGs are structural analogues of prostaglandin F_2α_ and they bring about their action by binding to prostaglandin F receptor. The vast majority of the study subjects on PG were using bimatoprost in the study cohort. Further, study subjects using beta‐blockers were all on timolol.

### 2.2 RNA isolation and quantitative real‐time PCR

Total RNA was isolated from trabecular meshwork tissue and iris tissue using TRIzol method according to manufacturer's protocol (Invitrogen). The concentration and purity of the extracted mRNA was assessed, and samples that had at least 1000 ng of RNA with a purity by optical density 260/280 ratio of >1.6 were selected for further analysis. RNA was converted into cDNA using Bio‐Rad iSCRIPT cDNA conversion kit (Bio‐Rad). Real‐time PCR was performed using SYBR green reagent (Kapa Biosystems Inc). The quantitative real‐time PCR cycle includes pre‐incubation at 95°C for 3 minutes, 40 amplification cycles at 95°C for 10 seconds, 58°C for 30 seconds using a CFX Connect™ real‐time PCR detection system (Bio‐Rad). Primer sequence details are provided in Table S1. The expression levels of human *TGFβ1* (transforming growth factor beta 1), *TGFβ2* (transforming growth factor beta 2), TGFβR2 (transforming growth factor beta receptor 2), *CTGF* (connective tissue growth factor), *FN* (fibronectin), *LOXL2* (lysyl oxidase–like 2), *WNT3A* (wingless‐type family member 3A), *DECORIN* (DSPG2, dermatan sulphate proteoglycans II), *HEVIN* (SPARCL1, secreted protein acidic and rich in cysteine‐like 1) and *ADBR2* (β2‐adrenergic receptor) were determined by normalizing the expression of these genes to housekeeping gene, *β‐actin* in the respective samples. Normalized expression value of zero indicative of no detectable expression of the gene of interest in a particular sample was excluded from the analysis. Pro‐fibrotic genes studied include *TGFβ1*, *TGFβ2*, *TGFβR2*, *CTGF*, *FN*, *LOXL2* and *WNT3A*, and the anti‐fibrotic genes were *DECORIN* and *HEVIN*.

### 2.3 In vitro cell–based experiments

Human trabecular meshwork (hTM) cells (a kind gift from Prof. Donna Peters, University of Wisconsin‐Madison, WI, USA) were cultured in vitro in DMEM containing 10% FBS maintained at 37°C, 5% CO_2_. hTM cells (1 × 10^6^ cells/well of 6‐well plate) were treated either with prostaglandin analogue (bimatoprost 0.01% ophthalmic solution, Lumigan, Allergan), beta‐blocker (timolol maleate, 0.5% ophthalmic solution, Glucomol, Allergan) or the combination of both for 24 hours at a dilution of 1:100. Since bimatoprost and timolol were the most commonly used PG and BB in the study cohort, those were chosen for in vitro validation experiments. A dilution of 1:100 of PG and/or BB was chosen to study its effects on TM, because it is known that less than 5% of the instilled drug as an eye drop penetrates the ocular surface and reaches intraocular tissues including the anterior chamber.^29^, ^30^ Further, the hTM cells were treated with a selective inhibitor of TGFβ/SMAD signalling^31^ (SB431542, MedChemExpress LLC) 30 minutes prior to either recombinant human TGFβ1 (Merck KGaA) or recombinant human TGFβ2 (Merck KGaA) treatment for 2 hours. The viability of hTM cells at the end of treatment was determined by trypan blue exclusion assay. The cells were then harvested for RNA extraction and whole cell protein lysate preparation as described elsewhere in the method section. The expression of mRNA and protein of specific genes were measured as described elsewhere in the manuscript. The cell culture supernatants were also collected to measure the levels of TGFβ1 in them by bead‐based ELISA.

### 2.4 Immunoblotting

hTM cells following treatment as indicated earlier were lysed using RIPA buffer (20 mmol/L Tris pH 8.0, 0.1% SDS, 150 mmol/L NaCl, 0.08% sodium deoxycholate, 1% NP40 supplemented with protease inhibitor and phosphatase inhibitor) for 30 minutes. The clarified whole cell protein lysates (WCL) were obtained following centrifugation. Proteins in WCL (20 μg) for each sample were separated on 10% SDS‐PAGE (sodium dodecyl sulphate‐polyacrylamide gel electrophoresis) gel. The proteins were then transferred onto a PVDF (polyvinylidene difluoride) membrane followed by blocking at room temperature for an hour using 5% fat free milk diluted in TBST (Tris‐buffered saline with 0.1% Tween 20). Primary antibodies against FN (Abcam plc), CTGF (Abcam plc), decorin (Abcam plc), total SMAD3 (Abcam plc) and phosphorylated SMAD3 (Abgenex Pvt. Ltd) were used at a dilution of 1:1000 except for GAPDH (Abcam plc) which was used at 1:5000 in 5% fat free milk in TBST. The membranes were incubated with respective primary antibodies overnight at 4°C. The membranes were washed using TBST and incubated with the relevant secondary antibodies (antimouse and anti‐rabbit, BosterBio) at 1:5000 dilutions for an hour at room temperature. The membranes were then washed and incubated with Clarity ECL Western blotting substrate (Bio‐Rad) and resulting chemiluminescence was imaged using Image quant (GE Image Quant LAS 500, GE Healthcare). Densitometry analysis was done using ImageJ software (Version 6).

### 2.5 Cytometric bead array

The levels of TGFβ1 in hTM cell culture supernatants were measured by bead‐based ELISA^32^ (Cytometric Bead Array, BD^TM^ CBA Human Soluble Protein Flex Set System, BD Biosciences) using a flow cytometer (BD FACSCantoII, BD Biosciences). The beads and fluorescent signal intensities were acquired and recorded using BD FACSDiva software (BD Biosciences). Standards were used to determine the absolute concentration of TGFβ1 in the supernatants, and the calculation was performed using FCAP array Version 3.0 (BD Biosciences).

### 2.6 Statistical analysis

Statistical significance between the groups was determined by using unpaired *t* test or Mann–Whitney test based on the distribution of the data. Shapiro–Wilk normality test was used to determine the distribution type of the data. *P* < .05 was considered to be statistically significant. GraphPad prism version 6 (GraphPad Software, Inc) was used to perform statistical analysis.

## 3 RESULTS

A total of 32 POAG and 37 PACG patients were included in the study. The clinical characteristics as described in Table 1 show no significant differences in the parameters such as age, sex, IOP, MD, PSD and VFI between the two groups. The expression of fibrosis‐associated genes measured in the TM tissues exhibited no significant differences between POAG and PACG patients (Table 2). The overall cohort of primary glaucoma patients (POAG and PACG) was further divided based on the type of IOP‐lowering medication usage prior to surgery, and their clinical characteristics are listed in Table 3. All parameters among the groups based on IOP‐lowering medication type used were similar except for the age and duration of drug usage.

**TABLE 1:** Cohort characteristics of study subjects based on glaucoma types

	POAG (mean ± SEM; median)	PACG (mean ± SEM; median)	*P* value
Sample size	32	37	NA
Sex (M/F)	24/8	24/13	NA
Age (y)	64.25 ± 1.7; 65	64.24 ± 1.7; 68	.647^†^
IOP (mm Hg)	17.0 ± 0.7; 16.5	20.35 ± 1.2; 18	.088
MD	−20.06 ± 1.7; −20.7	−20.9 ± 1.5; −22.5	.698*
PSD	6.3 ± 0.5; 6.4	7.0 ± 0.5:7.3	.321*
VFI score	42.3 ± 6.1; 33.5	36.0 ± 5.2; 25	.494^†^
Duration of IOP‐lowering medications (months)	19.5 ± 3.9; 8	11.0 ± 2.5; 3.5	.213^†^

Abbreviations: IOP, intraocular pressure; MD, mean deviation; PACG, primary angle‐closure glaucoma; POAG, primary open‐angle glaucoma; PSD, pattern standard deviation; SEM, standard error of mean; VFI, visual field index.

* Unpaired *t* test.

^†^ Mann–Whitney test.

**TABLE 2:** Gene expression profile in trabecular meshwork tissue in the study cohort

	POAG (mean ± SEM; median)	PACG (mean ± SEM; median)	*P* value
*TGFβ1*	0.038 ± 0.022; 0.003	0.011 ± 0.003; 0.005	.7466
*TGFβ2*	0.053 ± 0.028; 0.007	0.076 ± 0.052; 0.006	.7567
*TGFβR2*	0.135 ± 0.075; 0.033	0.057 ± 0.027; 0.013	.2379
*CTGF*	0.546 ± 0.218; 0.016	0.684 ± 0.334; 0.016	.7661
*FN*	0.023 ± 0.005; 0.016	0.037 ± 0.011; 0.005	.4314
*LOXL2*	0.012 ± 0.005; 0.004	0.009 ± 0.002; 0.004	.7874
*WNT3A*	0.121 ± 0.030; 0.042	0.048 ± 0.012; 0.010	.0566
*HEVIN*	2.487 ± 1.353; 0.024	7.737 ± 3.377; 0.025	.7914
*DECORIN*	0.085 ± 0.035; 0.012	0.351 ± 0.219; 0.020	.7032

Note

Mann–Whitney test was used for statistical analysis.

Abbreviations: *CTGF*, connective tissue growth factor; *DECORIN*, *DSPG2*, dermatan sulphate proteoglycans II; *FN*, fibronectin; *HEVIN*, *SPARCL1*, secreted protein acidic and rich in cysteine‐like 1; *LOXL2*, lysyl oxidase–like 2; *PACG*, primary angle‐closure glaucoma; *POAG*, primary open‐angle glaucoma; SEM, standard error of mean; *TGFβ1*, transforming growth factor beta 1; *TGFβ2*, transforming growth factor beta 2; *TGFβR2*, transforming growth factor beta receptor 2; *WNT3A*, wingless‐type family member 3A.

**TABLE 3:** Cohort characteristics of study subjects based on IOP‐lowering medications

	PG (mean ± SEM; median)	BB (mean ± SEM; median)	PG+BB (mean ± SEM; median)	*P* value (PG vs BB)	*P* value (PG vs PG+BB)	*P* value (BB vs PG+BB)
Sample size	14	28	27	NA	NA	NA
Sex (M/F)	7/7	23/5	18/9	NA	NA	NA
Age (y)	70.9 ± 1.2; 72	61.2 ± 1.7; 62	63.9 ± 2.2; 66	.0006*	.0357*	.3362*
IOP (mm Hg)	16.7 ± 0.9; 16	20.1 ± 1.3; 18	18.4 ± 1.3; 18	.2336^†^	.8328^†^	.3063^†^
MD	−15.4 ± 2.6; −14.6	−23.5 ± 1.5; −24.1	−20.57 ± 1.9; −23.4	.0069*	.1218^†^	.3447^†^
PSD	6.1 ± 0.9; 5.9	7.0 ± 0.5:7.2	6.7 ± 0.6:6.6	.3588*	.5933*	.6748*
VFI score	56.2 ± 8.1; 58	30.8 ± 5.3; 24	37.3 ± 6.7; 24	.0173^†^	.0576^†^	.8996^†^
Duration of IOP‐lowering medications (months)	16.3 ± 4.9; 7.3	4.8 ± 1.4; 1.6	24.6 ± 4.3; 22	.0512^†^	.244*	<.0001^†^

Abbreviations: BB, beta‐blockers; IOP, intraocular pressure; MD, mean deviation; PG, prostaglandin analogues; PSD, pattern standard deviation; SEM, standard error of mean; VFI, visual field index.

* Unpaired *t* test.

^†^ Mann–Whitney test.

**FIGURE 1:**
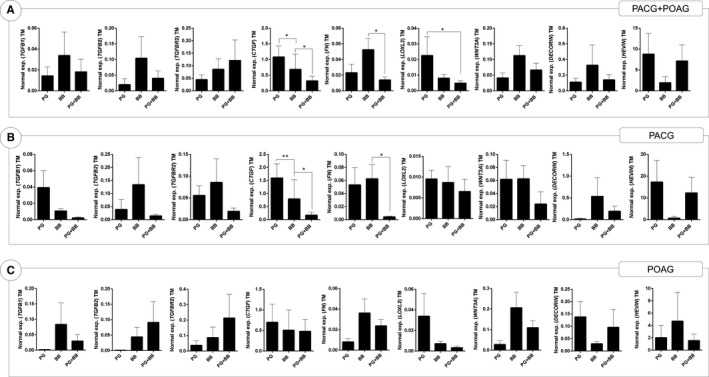
The effect of prostaglandin analogues or beta‐blockers alone and in combination on the differential gene expression of fibrosis‐associated genes in trabecular meshwork tissue of primary glaucoma patients. Graphs indicate mean mRNA expression of *TGFβ1*, *TGFβ2*, *TGFβR2*, *CTGF*, *FN*, *LOXL2*, *WNT3A*, *DECORIN* and *HEVIN *normalized to expression of *β‐ACTIN* (housekeeping gene). The categories include TM from primary glaucoma patients using prostaglandin analogues (PG), beta‐blockers (BB) or combination of prostaglandin analogues and beta‐blockers (PG+BB). Panel A indicates gene expression profile in trabecular meshwork tissue of primary glaucoma patients (primary angle‐closure glaucoma—PACG and primary open‐angle glaucoma—POAG). Panel B indicates gene expression profile in trabecular meshwork tissue of PACG patients. Panel C indicates gene expression profile in trabecular meshwork tissue of POAG patients. Bar graphs represent the mean ± SEM of all patients. *P < .05, **P < .01, Mann–Whitney test

The expression of the fibrosis‐associated genes in the TM tissue of primary glaucoma patients was evaluated on the basis of IOP‐lowering medication usage (PG, BB and PG+BB combination). The expression of pro‐fibrotic genes such as *TGFβ1*, *TGFβ2*, *CTGF*, *FN*, *LOXL2* and *WNT3A* was observed to be lower in PG+BB group as compared to either PG or BB alone (Figure 1). Statistically significant (*P* < .05) changes were observed only in *CTGF*, *LOXL2* and *FN* genes (Figure 1A). In addition, the expression of CTGF was observed to be significantly lower in BB compared to PG (Figure 1A). Anti‐fibrotic genes, *DECORIN* and *HEVIN* did not show any significant or distinct pattern related to medication usage. In order to minimize individual specific variations in expression of the genes in TM among the patients due to the individual genetic or epigenetic characteristics, the gene expression in TM tissue was normalized to that of the respective iris (TM/iris ratio) in each patient. The expression levels in iris tissue were not expected to be affected due to the disease. No distinct expression pattern of fibrosis‐associated genes studied was observed in the iris of primary glaucoma patients (data not shown). TM/iris expression ratio (Figure S1) of the genes studied showed similar normalized expression patterns as observed in TM tissue (Figure 1A). The gene expressions were further analysed separately in TM tissues from PACG and POAG patients. TM from PACG patients demonstrated significant differences in *CTGF* and *FN *(Figure 1B). Nevertheless, the TM tissues from PACG patients exhibited reduced expression pattern of pro‐fibrotic genes in those using PG+BB as compared those using PG or BB (Figure 1B) alone, a pattern similar to that observed in TM tissue of primary glaucoma patients (Figure 1A). The expression of genes in TM of PACG patients normalized to the expression of respective iris tissue (TM/iris ratio) is shown in Figure S2. The expression of *TGFβ1*, *CTGF*, *FN*, *LOXL2* and *WNT3a* in TM tissues of POAG patients was observed to be lower in patients on PG+BB, rather than those on either PG or BB (Figure 1C). Similar trend in the expression pattern of the genes was observed when the expression of these genes in TM was normalized to the expression of matched iris—TM/iris ratio in POAG patients (Figure S3).

**FIGURE 2:**
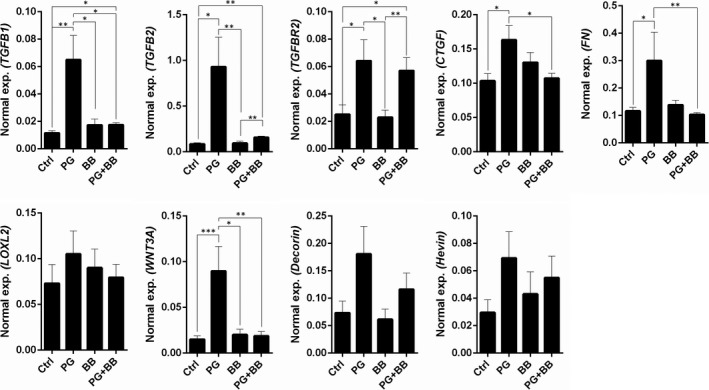
The effect of prostaglandin analogues or beta‐blockers alone and in combination on the differential gene expression of fibrosis‐associated genes in human trabecular meshwork cells in vitro. Graphs indicate mean mRNA expression of *TGFβ1*, *TGFβ2*, *TGFβR2*, *CTGF*, *FN*, *LOXL2*, *WNT3A*, *DECORIN* and *HEVIN* normalized to expression of *β‐ACTIN* (housekeeping gene) in human TM cells in vitro following IOP‐lowering medication for 24 h. The categories include untreated controls (Ctrl), prostaglandin analogue (PG), beta‐blocker (BB) or combination of prostaglandin analogue and beta‐blocker (PG+BB) treatments. Bar graphs represent the mean ± SEM of six independent experiments. *P < .05, **P < .01, ***P < .001, Mann‐Whitney test

These observations suggest that the use of PG+BB, rather than the use of either PG or BB, is associated with reduced fibrotic status in TM of primary glaucoma patients. The causal role of this association was investigated by treating trabecular meshwork cells, in vitro, with either PG or BB or PG+BB and subsequently by measuring the expression of the fibrosis‐associated genes. We observed that the mRNA expression of TGFβ1*, *TGFβ2*, CTGF*, FN*, *LOXL2* and WNT3a* was lower in the group of hTM cells treated with PG+BB combination rather than with either PG or BB (Figure 2; *P < .05). The results also demonstrate that PG induced expression of TGFβ1*, TGFβ2*, TGFβR2*, CTGF*, FN*, *LOXL2*, WNT3a*, *DECORIN* and *HEVIN* compared to untreated controls as shown in Figure 2 (*P < .05). On the contrary, BB did not induce any marked increase in the expression of the genes studied (Figure 2). Further, the combination of PG+BB was observed to inhibit the PG‐induced expression of these genes. It is important to note that the combination of PG+BB increased the expression of anti‐fibrotic genes, *DECORIN* and *HEVIN*, though not significantly, in hTM cells (Figure 2). The viability of hTM cells was not affected following exposure to these drugs (Figure S4).

**FIGURE 3:**
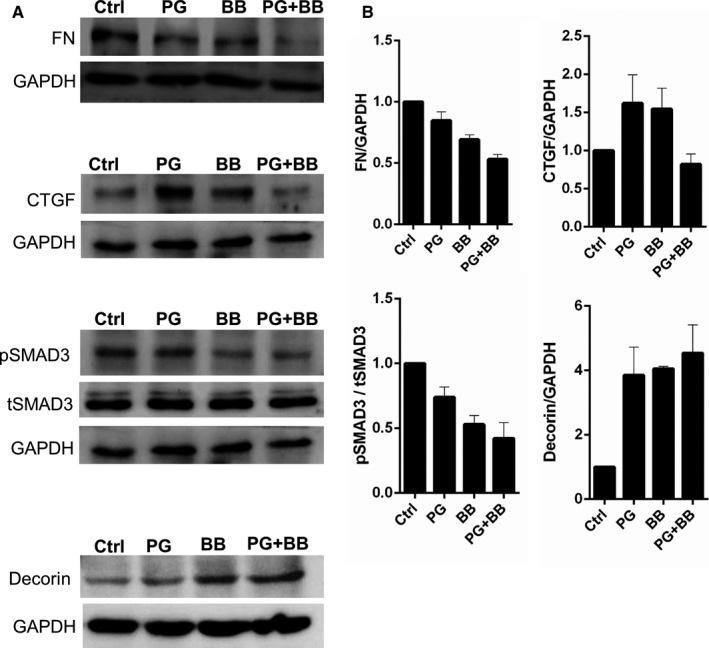
Protein validation of prostaglandin analogues or beta‐blockers alone and in combination on the differential gene expression of fibrosis‐associated genes in human trabecular meshwork cells in vitro. Panel A shows protein expression validation by immunoblotting for FN, CTGF, pSMAD3 (phosphorylated SMAD3), tSMAD3 (total SMAD3) and decorin following treatment of TM cells with IOP‐lowering medications for 24 h in vitro. The categories include untreated controls (Ctrl), prostaglandin analogue (PG), beta‐blocker (BB) or combination of prostaglandin analogue and beta‐blocker (PG+BB) treatments. GAPDH was used as protein loading controls. The blots shown are representative images of three independent experiments. Panel B exhibits quantification of protein expression of the immunoblot by densitometry analysis. The expression of the protein is indicated as ratio of respective protein to GAPDH (indicated in the y‐axis). The expression of pSMAD3 was quantified by normalizing its level to total SMAD3 expression (indicated in the y‐axis). Bar graphs represent the mean ± SEM of three independent experiments

These mRNA expression observations were further validated by measuring the protein levels of FN, CTGF, decorin and TGFβ‐specific signalling via the phosphorylation of SMAD3 by immunoblotting in hTM cells treated with PG, BB or PG+BB. Figure 3 indicates that the expression of FN was lower in hTM cells treated with PG+BB compared to either PG or BB and controls. The level of CTGF was observed to be higher following PG or BB treatment, but was similar to that of the controls in cells treated with PG+BB (Figure 3). Phosphorylation of SMAD3 is a key event in the intracellular signalling cascade induced by TGFβ (ligand) interaction with TGFβ receptor. We also observed a marked reduction in the basal levels of phosphorylated SMAD3 in hTM cells treated with PG+BB compared to either PG, BB or controls (Figure 3). Basal level of phosphorylated SMAD3 observed in cells is known to be due to constitutive TGFβ‐induced signalling. This was confirmed with the presence of TGFβ1 in the cell culture supernatants of hTM cells (Figure S5A). Further, basal and TGFβ1/2‐induced phosphorylated SMAD3 were reduced by SB431542, a selective TGFβ signalling inhibitor (Figure S5B,C). It is noteworthy that the levels of TGFβ in the cell culture supernatants of hTM cells were not altered by PG, BB or PG+BB treatment; nonetheless, a reduction in the basal levels of phosphorylated SMAD3 in hTM cells treated with PG+BB compared to either PG, BB or controls was observed. Another notable finding is that the decorin levels were observed to be increased by IOP‐lowering medication treatment, more so by PG+BB compared to controls (Figure 3A). These observations indicate the influence of IOP‐lowering medications on TGFβ‐mediated cellular response in hTM cells and also confirm the plausible causal role of PG+BB in reducing the pro‐fibrotic status in TM tissue in glaucoma patients.

## 4 DISCUSSION

Impediment to the aqueous humour out‐flow mechanisms in primary glaucoma, either in POAG or chronic PACG, is related to obstruction of the conventional out‐flow pathways.^33^ Aberrant ECM deposition in TM serves as a primary aetiopathological factor in POAG, while in PACG, it occurs secondary to physical apposition of cornea and iris angle. This eventually leads to pore closure, increased resistance to aqueous drainage and raised IOP.^33^ A number of pro‐fibrotic factors including TGFβ2 and CTGF have been reported to have a role in the initiation of pore closure in human TM.^6^, ^34^, ^35^ There is no directed therapy to mitigate glaucoma progression by reversing or halting the fibrosis‐associated pore closure in TM. There is emerging evidence, regarding this possibility by the use of Rho kinase inhibitors which are reported to improve aqueous out‐flow by favourably modulating fibrosis‐associated factors including CTGF in TM.^36^, ^37^ However, the status of such pro‐fibrotic pathways in human glaucomatous trabecular meshwork is not yet understood.

The conventional treatment modalities that involve medical therapy are not oriented towards the primary pathology, that is changes in TM architecture. Most of the drugs are thought to function primarily by suppression of aqueous humour production like beta‐blockers, carbonic anhydrase inhibitors and alpha agonists; or improving aqueous out‐flow by uveo‐scleral or alternate pathways like prostaglandin analogues and adrenergic drugs.^11^, ^12^ Hence, the current methods of treatment revolve around decreasing the IOP without addressing the natural conventional out‐flow pathway, which contributes to ~90% of aqueous drainage.^2^

Glaucoma being a progressive disease, it is important to address the increasing out‐flow resistance by conventional pathway in addition to lowering the IOP by alternate means. Further, it is important to understand whether the natural out‐flow mechanisms are affected by the treatment with current modalities. Therefore, instead of finding new drugs for inhibition of the fibrotic pathways, it would beneficial to explore whether existing IOP‐lowering medications have such properties. Hence, the current study investigated the effect of the most commonly used IOP‐lowering medications, that is PG, BB and their combination (PG+BB) on fibrosis‐associated factors in TM of primary glaucoma patients and hTM cells.

The current study demonstrates that the both PG and BB differentially affect the expression of fibrosis‐associated factors in human TM. Further, differential expression of fibrosis‐associated factors by PG+BB indicates a favourable effect to reduce fibrosis or pore closure factors in TM tissue of POAG and PACG patients. The effect was also substantiated by in vitro experiments on cultured hTM cells with PG+BB combination providing a favourable regulation of fibrosis‐associated genes. In vitro validation clearly showed that even 24‐hour exposure of the drug to the hTM cells altered the pro‐fibrotic protein levels and the upstream signalling factors.

Aberrant TGFβ response is known to contribute to fibrosis in general.^38^ Dysregulation in the factors regulating ECM remodelling has been implicated in TM fibrosis.^10^ TGFβ produced by hTM cells is also reported to regulate the pore closure mechanisms in human TM.^10^ TGFβ mediates its effects by induction of CTGF, FN, collagens and plasminogen activator inhibitor.^39^, ^40^ Hence, aberrant TGFβ expression or signalling in TM would result in abnormal TM architecture.^41^, ^42^ TGFβ2 was reported to be higher in aqueous humour of glaucoma patients.^34^ Hence, it would be ideal to block TGFβ‐induced signalling and responses in TM to reduce the resistance to conventional out‐flow system. TGFβ‐induced intracellular signalling cascade involves activation of SMAD2/3.^39^ The in vitro experiments demonstrate that the PG+BB combination inhibits phosphorylation of SMAD3. This suggests that the reduction in the basal expression of TGFβ‐dependent fibrosis‐associated factors such as FN and CTGF by PG+BB could be due to the inhibition of the TGFβ signalling cascade. However, further resolution of this mechanism underpinning these drug‐induced cellular signalling events is necessary. Similar studies are required to investigate the effect of other PG analogues and IOP‐lowering medications on TM fibrosis, since, various PG analogues in glaucoma management have been reported to have differential effects on ECM remodelling.^24^, ^43^, ^44^ In addition, possibly due to reduction in pro‐fibrotic factors, an increase in the expression of decorin by PG+BB was also observed, in vitro. Decorin has been reported to have anti‐fibrotic functions and have been shown to resolve fibrosis in various systems including TM and cornea.^45^, ^46^, ^47^

The role of beta‐adrenergic signalling in driving TGFβ expression and fibrosis‐associated changes in cardiac tissues has been reported.^48^, ^49^ Beta‐blockers have been shown to reduce pro‐fibrotic mediators and fibrotic changes in liver and cardiac tissues in animal models.^50^, ^51^, ^52^ TM tissues have been shown to express beta‐adrenergic receptors^53^, ^54^ and was observed to be present in hTM cells and TM tissues from the current study cohort (Figure S6). This suggests the plausibility of beta‐blockers regulating fibrosis‐associated mechanisms in the TM. Catecholamines are key ligands that bind and activate beta‐adrenergic receptors. Catecholamines are known to be present in aqueous humour in humans and have been reported to be elevated in glaucoma.^55^, ^56^, ^57^ Beta‐blockers could possibly be inhibiting the effect of aqueous humour catecholamines on the TM beta‐adrenergic receptors to reduce fibrosis‐associated changes. Despite the availability of knowledge regarding the role of prostaglandins in regulating fibrosis, there is paucity in details pertaining to inter‐regulation between prostaglandins and beta‐adrenergic receptor‐mediated responses. Further, it would be worthy to investigate the crosstalk between prostaglandin receptor and beta‐adrenergic receptor activation in driving fibrosis, which is yet to be explored. This understanding would be pivotal in unravelling the mechanism underlying PG+BB‐mediated reduction in pro‐fibrotic responses in TM.

The findings from this study provide a compelling insight into the possible effect of PG and/or BB on TM. The limitations of the study are the small sample size and small number of genes tested for their expression in the patient TM. However, the amount of TM tissue obtained during surgery is minute, precluding the testing of large numbers of genes. In addition, the study also did not have a healthy TM control group (TM tissues without known history of exposure to IOP‐lowering medications studied), since such invasive samples cannot be obtained from healthy eyes. Therefore, the findings observed in the patients were further validated in hTM cells, in vitro. Longitudinal studies including imaging of TM and/or ultrastructural studies using electron microscopy would provide structural evidence to the observations made. In addition, since our data provide evidence of modulation of gene expression patterns at the TM, future studies using RNA‐seq or proteomic methods are warranted. In addition to the known improved IOP‐lowering efficiency by the combination of PG and BB, our data provide additional molecular evidence that they also render fibrosis‐reducing effects in TM.

## CONFLICT OF INTEREST

The authors have no financial disclosures or conflicts of interest to declare pertaining to the study.

## AUTHOR CONTRIBUTIONS

ST contributed towards data acquisition, data interpretation and manuscript preparation. PM contributed towards data acquisition, data interpretation and manuscript preparation. APN contributed towards research design, data acquisition and analysis, and AG contributed towards research design and manuscript preparation. RKD contributed to data analysis and manuscript preparation. ASG contributed to research design, data interpretation and manuscript preparation. SS contributed towards research design, data analysis, data interpretation and manuscript preparation. All authors read and approved the final manuscript.

## Supporting information

Figure S1

Figure S2

Figure S3

Figure S4

Figure S5

Figure S6

Table S1

## Data Availability

The data that support the findings of this study are available on request from the corresponding author. The data are not publicly available due to privacy or ethical restrictions.
