# Supplementary figures and images for: Treatment of glaucoma by prostaglandin agonists and beta‐blockers in combination directly reduces pro‐fibrotic gene expression in trabecular meshwork
Source: J Cell Mol Med. 2020 Apr 8;24(9):5195–204. doi: 10.1111/jcmm.15172 (PMC7205793; doi:10.1111/jcmm.15172)

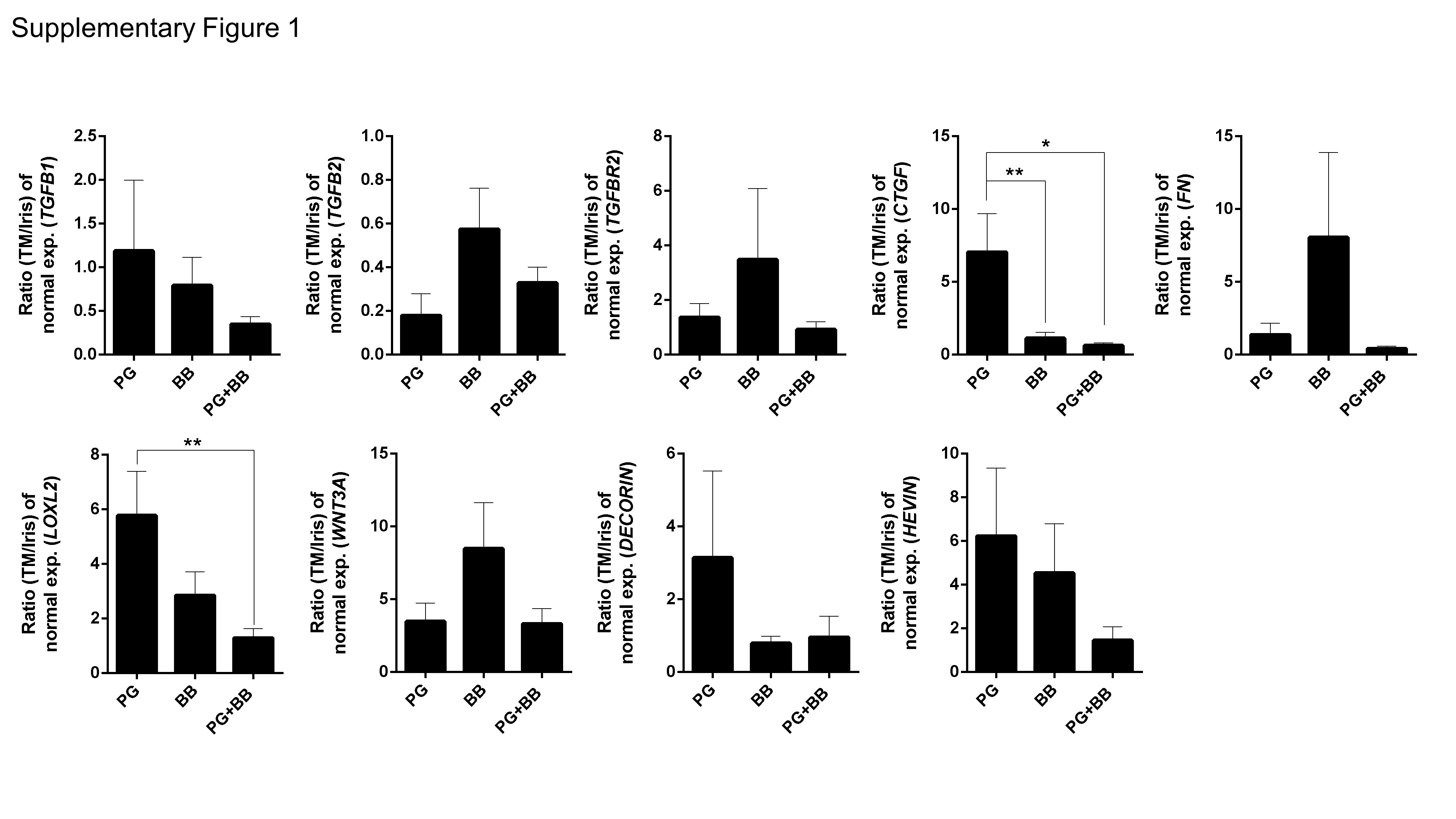

Supplement: Supplementary file 1 — Figure S1 [file JCMM-24-5195-s001.tif]

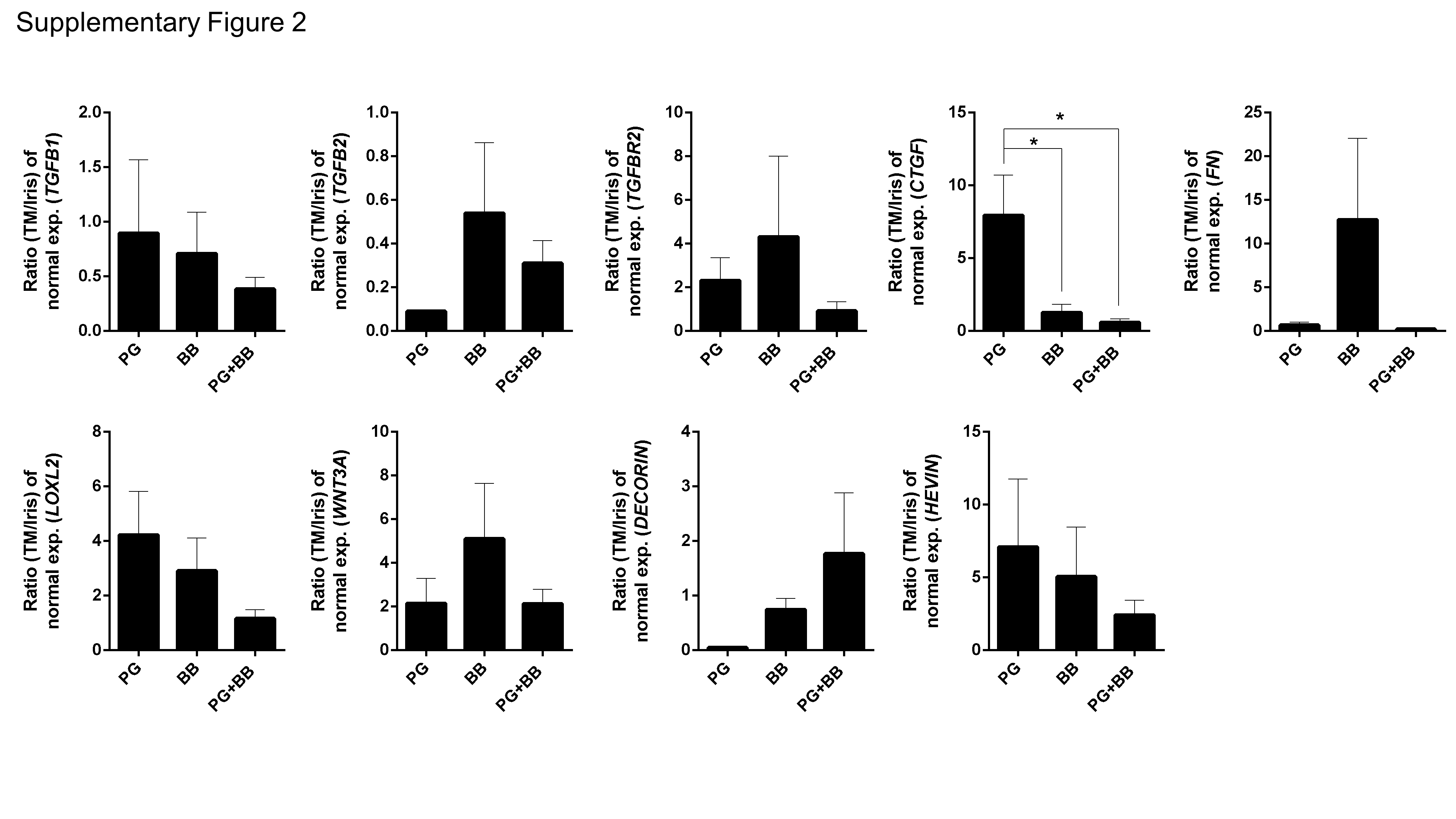

Supplement: Supplementary file 2 — Figure S2 [file JCMM-24-5195-s002.tif]

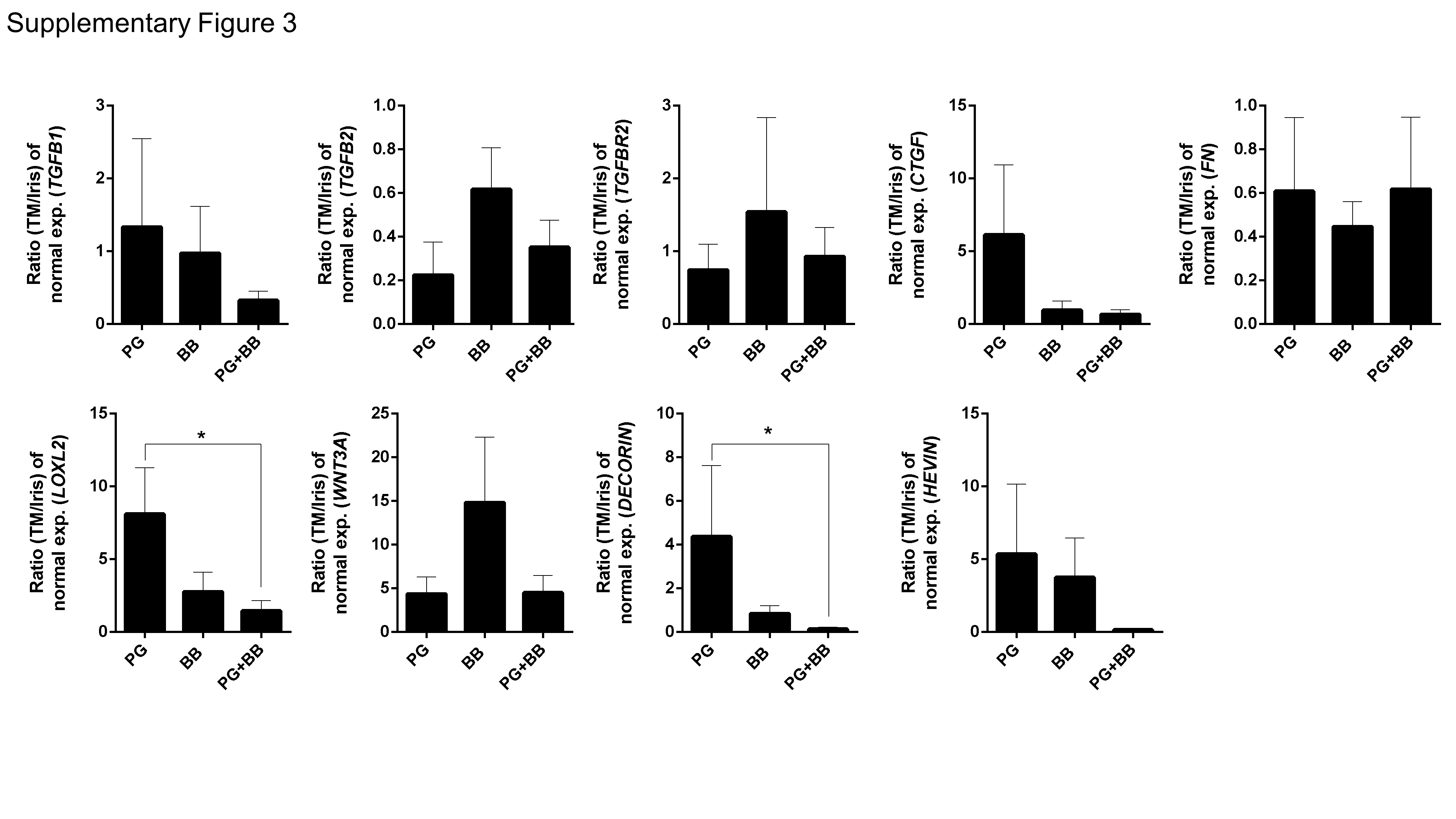

Supplement: Supplementary file 3 — Figure S3 [file JCMM-24-5195-s003.tif]

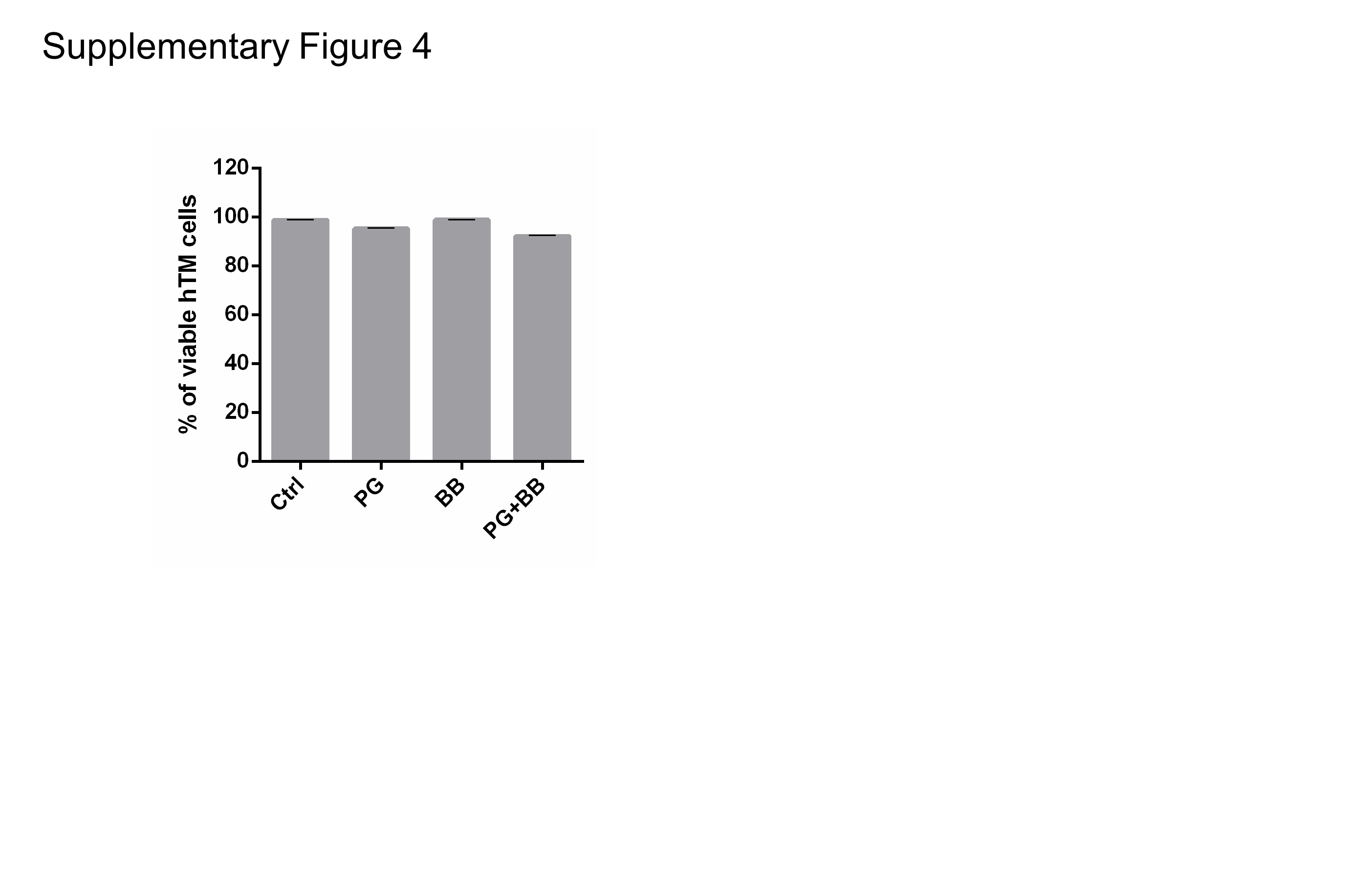

Supplement: Supplementary file 4 — Figure S4 [file JCMM-24-5195-s004.tif]

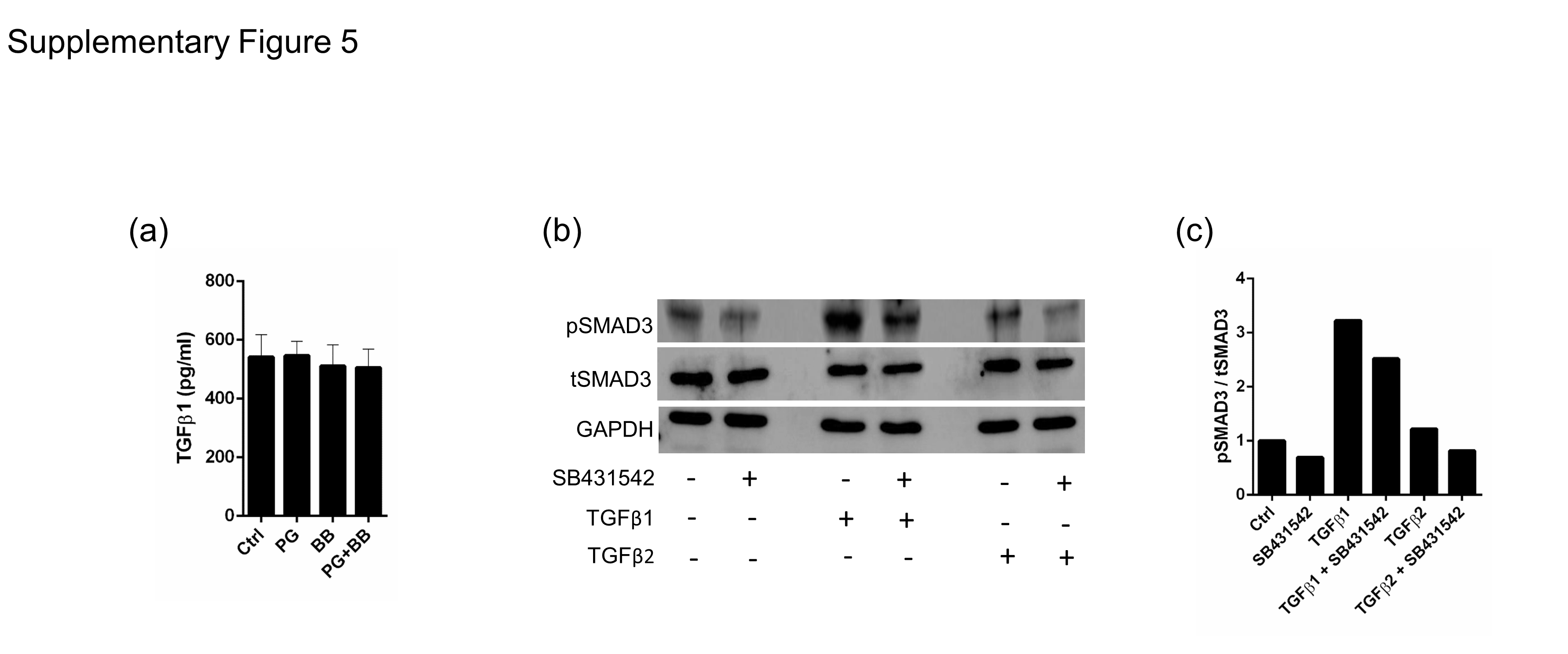

Supplement: Supplementary file 5 — Figure S5 [file JCMM-24-5195-s005.tif]

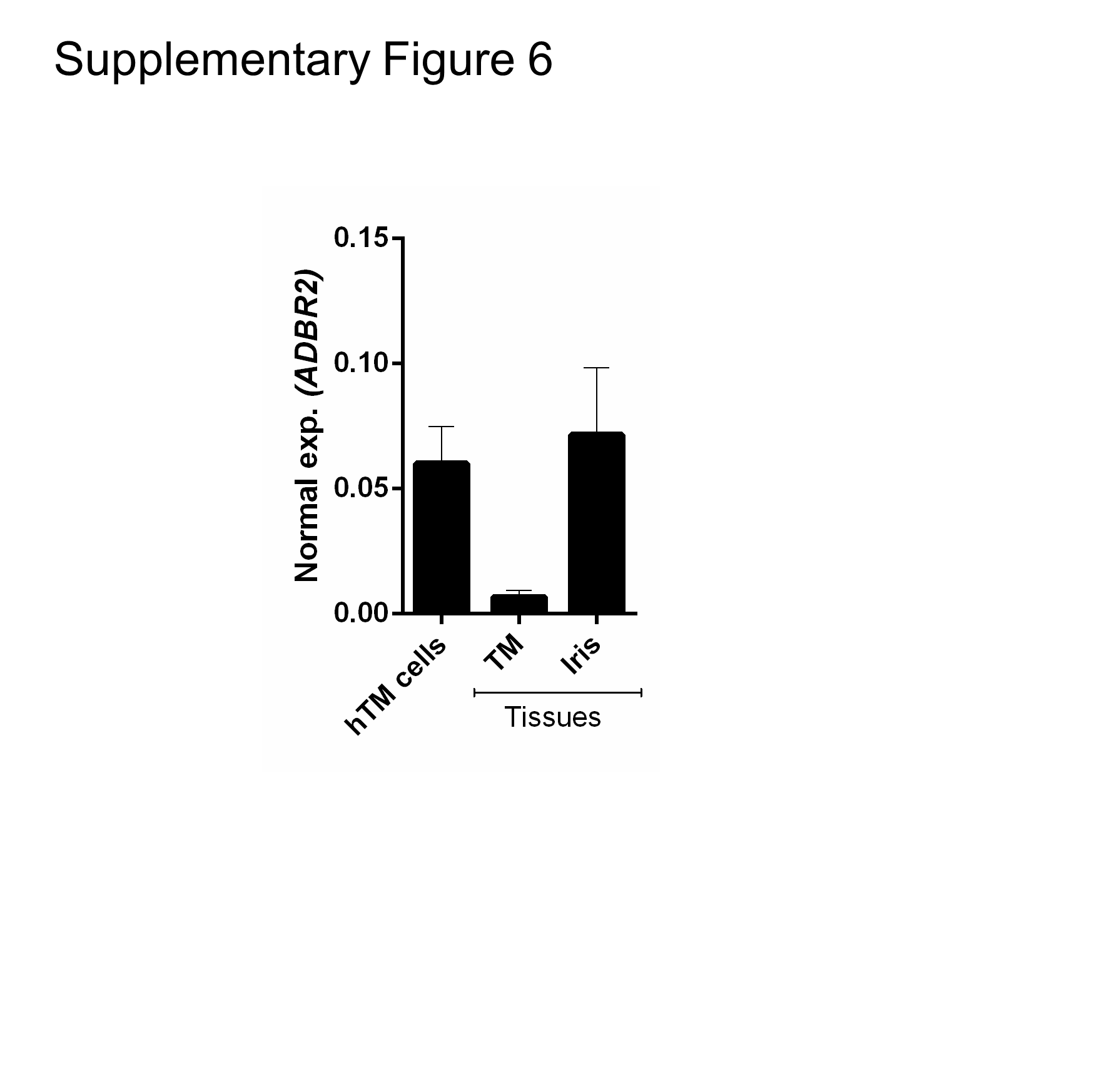

Supplement: Supplementary file 6 — Figure S6 [file JCMM-24-5195-s006.tif]
