# Supplementary material for: Treatment of glaucoma by prostaglandin agonists and beta‐blockers in combination directly reduces pro‐fibrotic gene expression in trabecular meshwork
Source: J Cell Mol Med. 2020 Apr 8;24(9):5195–204. doi: 10.1111/jcmm.15172 (PMC7205793; doi:10.1111/jcmm.15172)
Supplement: Supplementary file 7 — Table S1 [file JCMM-24-5195-s007.docx]

**Supplementary Table 1: Details of primers used in the study**

| **Gene Name** | **Forward Primer** | **Reverse Primer** |
| --- | --- | --- |
| **TGFβI** | CAGCAACAATTCCTGGCGATACCTC | CAACCACTGCCGCACAACTC |
| **TGFβ2** | AAGAGCAGAAGGCGAATGGC | AGTGCAGCAGGGACAGTGTA |
| **TGFβR2** | CGTGTGCCAACAACATCAAC | TGCTTCAGCTTGGCCTTATAG |
| **CTGF** | GACTGGAAGACACGTTTGGC | GCGTTGTCATTGGTAACCCG |
| **FN** | TGGCCAGTCCTACAACCAGTA | CTCGGGAATCTTCTCTGTCAGC |
| **LOXL2** | GCTTCTGCTTGGAGGACACA | GTCGATGTCATGGCGGTACA |
| **Wnt3A** | CATGAACCGCCACAACAAC | GACCACCAGCATGTCTTCA |
| **Decorin** | GCTTCTTATTCGGGTGTGAGT | TTCCGAGTTGAATGGCAGAG |
| **Hevin** | AGGGTGCATGCTGTGGATTC | CTGGCAGACACAGTGAGGTT |
| **ADBR2** | GCCTGTGCTGATCTGGTCAT | AATGGAAGTCCAAAACTCGCA |
| **β-actin** | GCCAACCGCGAGAAGATGA | CCATCACGATGCCAGTGGTA |
